# Spatial and Temporal Non-Uniform Changes in Left Ventricular Myocardial Strain in Dogs with Duchenne Muscular Dystrophy

**DOI:** 10.3390/jcdd10050217

**Published:** 2023-05-16

**Authors:** Bijan Ghaleh, Inès Barthélemy, Lucien Sambin, Alain Bizé, Daphné Corboz, Luc Hittinger, Stéphane Blot, Jin Bo Su

**Affiliations:** 1 Inserm U955-IMRB, Team 3, UPEC, Ecole Nationale Vétérinaire d’Alfort, 94700 Maisons-Alfort, France; bijan.ghaleh@inserm.fr (B.G.); lucien.sambin@inserm.fr (L.S.); alain.bize@inserm.fr (A.B.); daphne.corboz@inserm.fr (D.C.); luc.hittinger@aphp.fr (L.H.); 2 Assistance Publique—Hôpitaux de Paris, Hôpital Henri Mondor, Service de Cardiologie, 94010 Créteil, France; 3 Inserm U955-IMRB, Team10, UPEC, Ecole Nationale Vétérinaire d’Alfort, 94700 Maisons-Alfort, France; ines.barthelemy@vet-alfort.fr (I.B.); stephane.blot@inserm.fr (S.B.)

**Keywords:** canine model of duchenne muscular dystrophy, dystrophin-deficient cardiomyopathy, heterogeneity, myocardial strain, speckle-tracking echocardiography

## Abstract

Background: Understanding and effectively treating dystrophin-deficient cardiomyopathy is of high importance for Duchenne muscular dystrophy (DMD) patients due to their prolonged lifespan. We used two-dimensional speckle tracking echocardiography to analyze more deeply the non-uniformity of myocardial strain within the left ventricle during the progression of cardiomyopathy in golden retriever muscular dystrophy (GRMD) dogs. Methods: The circumferential strain (CS) and longitudinal strain (LS) of left ventricular (LV) endocardial, middle and epicardial layers were analyzed from three parasternal short-axis views and three apical views, respectively, in GRMD (n = 22) and healthy control dogs (n = 7) from 2 to 24 months of age. Results: In GRMD dogs, despite normal global systolic function (normal LV fractional shortening and ejection fraction), a reduction in systolic CS was detected in the three layers of the LV apex but not in the LV middle-chamber and base at 2 months of age. This spatial heterogeneity in CS progressed with age, whereas a decrease in systolic LS could be detected early at 2 months of age in the three layers of the LV wall from three apical views. Conclusions: Analyzing the evolution of myocardial CS and LS in GRMD dogs reveals spatial and temporal non-uniform alterations of LV myocardial strain, providing new insights into the progression of dystrophin-deficient cardiomyopathy in this relevant model of DMD.

## 1. Introduction

Duchenne muscular dystrophy (DMD) is an X-linked hereditary disease affecting approximately 1 in 5000 male births worldwide and is caused by mutations in the gene that encodes the 427-kDa cytoskeletal protein dystrophin. Dystrophin forms a dystrophin protein complex with dystrophin-associated proteins including dystroglycans, sarcoglycans, integrins and caveolin in the sarcolemma, linking the internal cytoskeleton to the extracellular matrix in striated muscle and playing an important role in stabilizing the cell membrane and signal transduction during muscle contraction [1]. The vast majority of DMD patients lack the dystrophin protein, making the membrane susceptible to damage by mechanical stress which can lead to muscle degeneration. DMD is characterized by progressive muscle degeneration and weakness. Beginning as early as 2 to 3 years of age, the principal symptom of DMD is muscle weakness, affecting the proximal muscles first, then the distal limb muscles and usually the lower external muscles before the upper external muscles. Generally, DMD patients are wheelchair-bound by 12 years of age and die of respiratory failure in their late teens or early twenties. Although there is currently no effective treatment for this disease, patients with DMD live significantly longer thanks to improved respiratory ventilation and other supportive care. As a result, deaths from heart failure rather than respiratory failure have increased [2]. Therefore, understanding the development of dystrophin-deficient cardiomyopathy and effectively treating it has become very important for DMD patients.

Similar to DMD, golden retriever muscular dystrophy (GRMD) is also caused by the dystrophin gene mutation, a single nucleotide change that leads to exon skipping and an out-of-frame DMD transcript [3]. GRMD dogs develop symptoms and signs like DMD patients and are used to explore pathological changes and test novel therapeutic strategies for DMD [4,5,6,7,8,9,10,11].

Using image-processing algorithms for two-dimensional digital echocardiographic images, two-dimensional speckle tracking echocardiography (2D-STE) identifies small stable myocardial footprints called speckles generated by ultrasound-myocardial tissue interactions within a defined region of interest and tracks speckles frame-to-frame over the cardiac cycle. Distances between speckles or their spatiotemporal displacement provide information about global and segmental myocardial deformation. Despite some technical factors influencing strain value [12], 2D-STE has been developed into a reliable method to objectively quantify left ventricular (LV) regional and global myocardial function [12,13] since strain information is independent of the Doppler angle of incidence [13]. Studies have shown that 2D-STE can be used to reveal early pathological changes in the heart of DMD patients [14,15,16] and GRMD dogs and cardiac responses in GRMD therapeutic trials [8,9,10]. Interestingly, recent technological developments have enabled 2D-STE to analyze LV wall strain in more detail, allowing, for example, the analysis of endocardial, middle and epicardial strains in different regions of the LV wall. Therefore, the aim of this study was to evaluate the changes in myocardial circumferential strain (CS) and longitudinal strain (LS) of LV endocardial, middle and epicardial layers in GRMD dogs aged from 2 to 24 months. These GRMD dogs were compared to healthy control dogs to reveal alterations in LV myocardial strain during the pathological development of dystrophin-deficient cardiomyopathy. Our results showed that spatial and temporal non-uniform alterations of myocardial strain occur within the left ventricle during the progression of the disease. This finding may provide insight into the progression of cardiomyopathy due to dystrophin deficiency in this highly relevant canine model of DMD.

## 2. Materials and Methods

### 2.1. Animal Model

Twenty-two GRMD and seven healthy control dogs were provided by the Centre d’Elevage du Domaine des Souches (Mezilles, France) and were cared for by veterinarians throughout the study. GRMD was diagnosed by DNA analysis at 1 month of age. The experimental protocol was approved by the animal ethics committee (ComEth ANSES-ENVA-UPEC agreement #20/12/12–18) and the experimental procedures were performed in accordance with the European Union Directive 2010/63/EU.

### 2.2. Echocardiographic Image Acquisition and Analysis

Echocardiographic image acquisition and analysis were performed by the same investigator in GRMD dogs and their healthy littermates at 2, 6, 9, 12, 18 and 24 months of age. Using a Vivid 7 ultrasound (General Electric Medical System, Horten, Norway), echocardiographic images were acquired from conscious (i.e., without the use of sedatives or anesthetics) 2-month-old puppies in a standing position using a 7S cardiac sector array probe and from dogs of other ages in a standing position in a sling using a 5S or an M4S cardiac sector array probe after an adaptive period of 20–30 min. The standing position allows for the acquisition of images from all commonly used LV views. An ECG was recorded during the echocardiographic examination to calculate heart rate.

M-mode parasternal short-axis (PSAX) images at the mid-chamber (papillary muscles) level were recorded to measure LV end-diastolic (EDD) and end-systolic (ESD) diameters, end-diastolic and end-systolic posterior and interventricular septal wall dimensions and fractional shortening (FS).

High-frame-rate videos (ranging from 60 to 119 frames/s) were recorded in apical 2-chamber, 3-chamber and 4-chamber views. In some cases, the apical 5-chamber view was used when an adequate 3-chamber image could not be obtained. The PSAX-base view was acquired at the level of mitral valve leaflets. The PSAX-mid-chamber view was recorded at the level of papillary muscles. The PSAX-apex view was recorded when the LV cavity was as small as possible without visible papillary muscles. For each view, a cine loop of at least three consecutive cardiac cycles was stored digitally for offline analysis. To ensure the image quality for strain analysis, the images of young dogs were generally acquired with the highest frame rate possible.

Echocardiographic images were analyzed as recommended [17]. FS (%) was calculated as [(LV EDD − LV ESD)/LV EDD × 100%]. LV end-diastolic volume (EDV, mL), end-systolic volume (ESV, mL) and ejection fraction (EF, %) were calculated by the biplane Simpson’s method using the apical 4-chamber and 2-chamber images.

Using dedicated software, 2D-STE was performed offline (EchoPac version 201, GE Healthcare, Horten, Norway). CS and LS were analyzed in endocardial (inner), middle and epicardial (outer) layers of GRMD and healthy control dogs of various ages using EchoPac version 201. The cardiac cycle length was measured from the beginning of systole. After delineating an area of interest within the LV wall and performing manual adjustments to include the entire LV wall, the software automatically divided the LV wall into 6 LV wall segments for each view. Each segment was divided into endocardial and epicardial parts and averaged to obtain endocardial, middle and epicardial strains. After viewing the validation results automatically generated by the software and adequately curve fitting the area of interest within the LV wall, the adequate tracking results were considered for analysis. It is often necessary to manually adjust the area of interest several times to obtain an adequate fitting. CS was analyzed in PSAX-apex, PSAX-mid-chamber and PSAX-base views. LS was analyzed in apical 3-chamber (or 5-chamber), 4-chamber and 2-chamber views.

### 2.3. Statistical Analysis

Continuous variables were expressed as mean ± SEM. Statistical analysis was performed with StatView (Version 5.0, Abacus Concepts Inc., Berkeley, CA, USA). The one-way ANOVA was used to test within-group differences over time (age). The ANOVA for repeated measurements over time was performed on echocardiographic data from 8 GRMD dogs and 4 healthy control dogs aged 2 to 24 months to test between-group differences. The two-tailed unpaired two-sample *t*-test was performed on data obtained in all dogs at each time point to determine the differences between GRMD and healthy control dogs. A difference was considered statistically significant at *p* < 0.05.

The assessment of inter-observer variability was not carried out in the study, because the acquisition and analysis of the echocardiographic images were carried out by a single investigator. To examine intra-observer variability, a set of images used for the calculation of EF, CS in the PSAX-apex view and LS in the 4-chamber view were taken from 6 randomly selected dogs used in the study and analyzed twice by the same sonographer in an interval of 24 h or more (3 heart beats were analyzed for each image). The interclass correlation coefficient (ICC) for these parameters was calculated using the Real Statistics Resource Pack. The coefficient of variation (CoV) for these parameters was calculated by the following equation: CoV (%) = standard deviation/mean × 100. The two measurements were used for the calculation of the ICC and the CoV for EF (0.908 and 3.1 ± 2.7%, respectively), endocardial CS (0.955 and −3.4 ± 2.9%), middle CS (0.941 and −3.8 ± 2.5%), epicardial CS (0.738 and −7.9 ± 5.7%), endocardial LS (0.958 and −3.1 ± 2.4%), middle LS (0.950 and −3.3 ± 2.7%) and epicardial LS (0.913 and −4.3 ± 4.2%).

## 3. Results

Of the 22 GRMD dogs included in the study, three were euthanized around the age of 6 months due to loss of ambulation; four between 9 and 12 months due to severely reduced mobility (n = 2), poor general conditions (n = 1) and severe bronchopneumonia (n = 1); three between 12 and 18 months of age due to reduced mobility (n = 2) and respiratory failure (n = 1); and two died of heart failure. No death occurred in healthy dogs throughout the study, and three of the seven healthy control dogs were rehomed after the 6-month examination.

GRMD dogs had significantly lower body weights than healthy control dogs at different ages (Table 1), notably reflecting a loss in muscle mass. In GRMD and healthy control dogs, the heart rate decreased with age, and the majority of GRMD dogs had slightly higher heart rates than healthy control dogs at the same age (Table 1).

### 3.1. Changes in LV Dimensional and Functional Parameters Measured by Echocardiography in GRMD and Healthy Control Dogs at Different Ages

As shown in Table 1, the LV EDD of healthy control dogs increased with age until 9 months old. The LV EDD of GRMD dogs increased with age but was smaller than that of healthy control dogs until 9 months of age. Thereafter, LV EDD was similar in both groups. LV ESD increased with age in healthy control and GRMD dogs. As a result, these changes in LV EDD and ESD of GRMD dogs resulted in a significant reduction in FS at 12, 18 and 24 months (*p* < 0.05 or *p* < 0.01). The decrease in FS in some GRMD dogs reached a pathological value (i.e., less than 28%) at 18 and 24 months, whereas healthy control dogs had a stable FS throughout all ages examined. In healthy control dogs, interventricular septal wall end-diastolic thickness (IVSWEDT) and LV posterior wall end-diastolic thickness (PWEDT) increased with age and reached a stable thickness at 12 months. In contrast, IVSWEDT and PWEDT of GRMD dogs were smaller than those of healthy control dogs starting from 6 and 9 months of age, respectively. However, interventricular septal wall systolic thickening (% IVSWTh) was not significantly changed over time in healthy control dogs and decreased, without reaching significance, in GRMD dogs. Similarly, LV posterior wall systolic thickening (% PWTh) did not significantly change throughout the protocol in healthy control dogs, whereas it significantly decreased over time in GRMD dogs.

LV end-diastolic volume (LVEDV) increased with age in both healthy control and GRMD dogs, but GRMD dogs had smaller LVEDV at each age examined, which is certainly due to the marked smaller size and body weight of GRMD dogs. LV end-systolic volume (LVESV) increased with age in healthy control dogs whereas GRMD dogs had a smaller LVESV at 2 and 6 months of age than healthy control dogs but a similar LVESV from 9 months of age. Consequently, GRMD dogs had smaller EF and, contrasted with healthy control dogs, EF decreased progressively with age, starting at 9 months whereas healthy control dogs had stable EF at all ages examined (Table 1). The decrease in EF in some GRMD dogs reached a pathological value (i.e., less than 50%) at 18 and 24 months old.

These data obtained by conventional echocardiography indicated that significant alterations in LV global function (FS and EF) can be detected rather tardily (12–18 months of age) in GRMD dogs.

### 3.2. Changes in CS in the Three LV Wall Layers Analyzed by 2D-STE from the Three Short-Axis Views in GRMD and Healthy Control Dogs through Time

**Figure 1:**
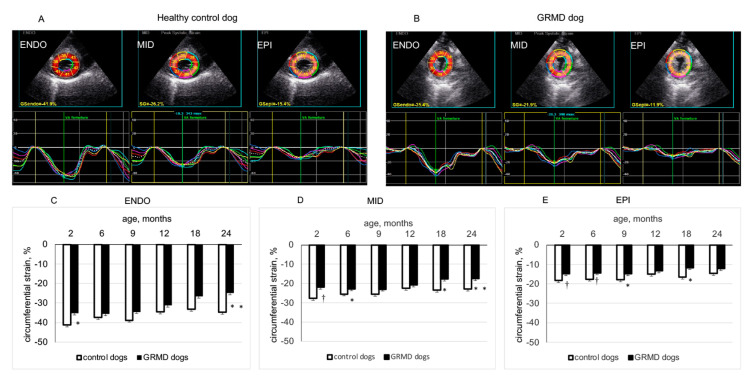
Circumferential strain (CS) of left ventricular (LV) endocardial (ENDO), middle (MID) and epicardial (EPI) layers analyzed from the parasternal short-axis-apex view. Panels (**A**,**B**) show the values and curves of 6 segments (showed by different colors) analyzed in each LV wall layer obtained in a 2-month-old healthy control dog and in an age-matched GRMD (golden retriever muscular dystrophy) dog. The curve in dotted line in each part of the LV wall is the average of the 6 curves. Panels (**C**–**E**) compare the peak systolic circumferential strain values of ENDO, MID and EPI layers of healthy control and GRMD dogs at different ages. Data were expressed as mean ± SEM. The one-way ANOVA was performed to test the within-group differences. The two-tailed unpaired two-sample *t*-test was performed on data obtained in all dogs at each time point to determine the difference between GRMD and healthy control dogs. n = 22, 22, 17, 13, 8 and 8 for GRMD dogs, and 7, 7, 4, 4, 4 and 4 for healthy control dogs at 2, 6, 9, 12, 18 and 24 months of age, respectively. * *p* < 0.05 and † *p* < 0.01 versus healthy control dogs of the same age.

Figure 1 shows two typical examples of LV endocardial, middle and epicardial CS from a 2-month-old healthy control dog (panel A) and a 2-month-old GRMD dog (panel B) analyzed by 2D-STE from the PSAX-apex view. As shown in Table 2, in the LV apical region, the peak CS of endocardial, middle and epicardial layers remained rather stable with age in healthy control dogs. In contrast, GRMD dogs showed significant reduction in peak CS in the three examined layers of the LV apex (values being less negative) at 2 months, which were mainly caused by the significantly smaller peak CS of LV anterior and antero-lateral regions than those of healthy control dogs. It is worth noting that this smaller peak CS of epicardial and middle layers in the LV apical region of GRMD dogs could be seen at the different ages examined.

At the PSAX-mid-chamber level, GRMD had similar peak CS in the endocardial layer as healthy control dogs except at 2 and 24 months of age and had smaller peak CS in epicardial and middle layers at 18 and 24 months of age. In the endocardial layer, this parameter significantly decreased with age in both groups. However, in the middle and epicardial layer, peak CS significantly decreased with age in GRMD dogs, whereas no significant change was detected in healthy control dogs. At the base of the left ventricle, the peak CS of endocardial, middle and epicardial layers was basically similar for GRMD and healthy control dogs except for the epicardial layer at 9, 18 and 24 months and the middle and endocardial layers at 24 months, where GRMD dogs had smaller peak CS than healthy control dogs. In the epicardial layer, basal peak CS decreased significantly with age in GRMD dogs, whereas this parameter remained stable over time in healthy control dogs.

These results indicated that GRMD dogs exhibited a non-uniform spatial alteration over time in CS, characterized by an early and persistent decrease in peak CS in the epicardial and middle layers of the LV apical region, mainly due to decreased peak CS in the apical anterior and antero-lateral regions. At the middle and basal levels of the left ventricle, decreased peak CS in GRMD dogs occurred later (at 18–24 months of age). In these regions of the left ventricle, the decrease in peak CS occurred earlier in epicardial and middle layers than in the endocardial layer.

### 3.3. Changes in LS in the Three LV Wall Layers Analyzed by 2D-STE from the Three Apical Views in GRMD and Healthy Control Dogs at Different Ages

**Figure 2:**
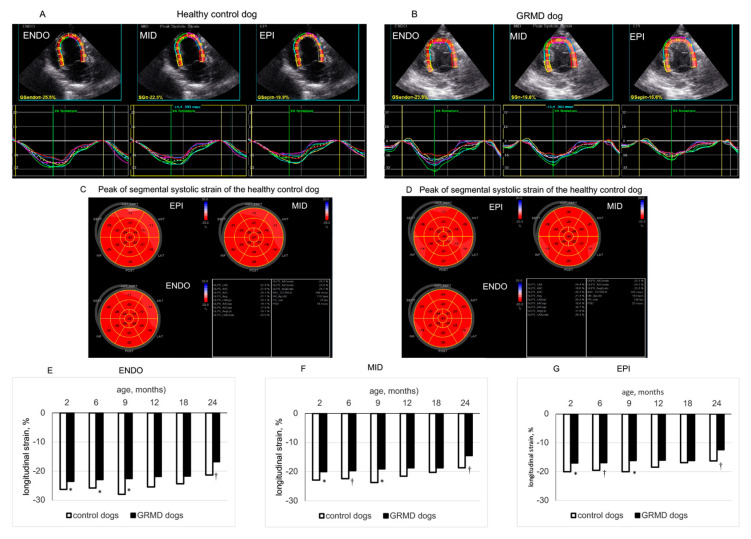
Longitudinal strain of left ventricular (LV) endocardial (ENDO), middle (MID) and epicardial (EPI) layers analyzed from the apical 4-chamber view. Panels (**A**,**B**) show the values and curves of 6 segments (showed by different colors) analyzed at each layer of the LV wall obtained in a 2-month-old healthy control dog and an age-matched GRMD (golden retriever muscular dystrophy) dog. The curve in dotted line in each part of the LV wall is the average of the 6 curves. Panels (**C**,**D**) are the bulls-eye presentations of segmental peak LS values obtained in each LV myocardial layer from 3 apical views of the above dogs. Panels (**E**–**G**) compare the longitudinal strain values of the ENDO, MID and EPI layers of healthy control and GRMD dogs at different ages. Data were expressed as mean ± SEM. The one-way ANOVA was performed to test within-group differences. The two-tailed unpaired two-sample t-test was performed on data obtained in all dogs at each time point to determine the differences between GRMD and healthy control dogs. n = 22, 22, 17, 13, 8 and 8 for GRMD dogs, and 7, 7, 4, 4, 4 and 4 for healthy control dogs at 2, 6, 9, 12, 18 and 24 months of age, respectively. * *p* < 0.05 and † *p* < 0.01 versus healthy control dogs of the same age.

Figure 2 shows two typical examples of LV endocardial, middle and epicardial LS from a 2-month-old healthy control dog (panel A) and a 2-month-old GRMD dog (panel B) analyzed by 2D-STE from the apical four-chamber view. As shown in Table 3, at the apical three-chamber view, GRMD dogs had significantly smaller peak LS than healthy control dogs at 2, 6, 9 and 12 months of age in the three layers of the LV wall. In the apical four-chamber view, peak LS of the three layers of LV wall were slightly but not significantly decreased with age in healthy control dogs. Contrastingly, GRMD dogs displayed a reduction with age and significantly smaller peak LS in the three layers of the LV wall at 2, 6, 9 and 24 months of age (Figure 2 and Table 3). In the apical two-chamber view, GRMD had significantly smaller peak LS in the endocardial layer than healthy control dogs at 2, 6, 9 and 12 months and significantly smaller peak LS in epicardial and middle layer at 2, 6 and 12 months (Table 3).

Thus, from a young age, a significantly smaller peak LS could be detected in the three layers of the LV wall of GRMD dogs from three apical views. The difference in peak LS between GRMD and healthy control dogs became less evident at 18 and 24 months of age.

## 4. Discussion

In this longitudinal study, significant alteration in global LV systolic function (i.e., decreased FS and EF) was revealed late by conventional echocardiography in GRMD dogs, as previously reported [6,8]. The decline in LV systolic function with age reflects the natural progression of cardiomyopathy in GRMD dogs in the absence of specific treatment targeting cardiomyopathy.

Myocardial regional mechanics can be assessed by echocardiography in terms of four principal types of strain or deformation: circumferential, longitudinal, radial and rotational. A combined analysis of these strains can give a complete description of LV myocardial strain. At present, simultaneously analyzing myocardial strains of LV endocardial, middle and epicardial layers by echocardiography is technically available for CS and LS. This study analyzed myocardial strain in terms of the CS and LS of different LV wall layers of different regions, with the hope that assessing LV myocardial strain from two aspects gives a more detailed description of the changes in LV myocardial regional strain during the development of cardiomyopathy in GRMD dogs. In fact, more detailed analysis of LV myocardial CS and LS revealed different change trends in LV myocardial strain in both the circumferential and longitudinal directions, showing earlier alterations consistent with previous findings [6,8]. More importantly, these alterations in LV function were not uniform within the LV wall, showing differential progression of cardiomyopathy from the apex to the base and from the epicardium to the endocardium.

Clinical studies using cardiac magnetic resonance image [18], tissue Doppler imaging (TDI) [19] and 2D-STE [16] showed that DMD boys (less than 10 years old) had a decreased peak CS which worsened with age. Interestingly, a study in DMD boys (aged 14.8 ± 3.1 years old) showed that peak CS in the posterior wall analyzed by TDI exhibited smaller values, more frequently, in the outer layer than in the inner layer [20]. In the present study, an analysis of endocardial, middle and epicardial CS by 2D-STE from PSAX-apex, PSAX-mid-chamber and PSAX-base views demonstrated a non-uniform change in peak CS in GRMD dogs, which occurred early (2 months of age) and manifested as a significant and persistently smaller peak CS in the epicardial and middle layers of the LV apical anterior and antero-lateral wall (Figure 2 and Table 2). Meanwhile, at the mid-chamber and basal levels of the left ventricle, a smaller CS was detected relatively later in GRMD dogs (Table 2). These results suggest that analysis of CS in the LV apical region may be a useful tool to reveal early manifestations of cardiomyopathy in GRMD dogs, paralleling the pattern described in DMD patients. This non-uniform distribution of functional alteration between the apical and basal areas was complemented by differential timing of reduced contractile function from the epicardial to the endocardial layer.

Previous studies have shown that a reduction in global systolic LS can be detected in young DMD boys [14,16,19,21] and GRMD dogs [8,10]. Going further, this study analyzed LS more deeply in the three layers of the LV wall from three apical views and showed that systolic LS was lower in the three LV wall layers in GRMD dogs than in healthy control dogs from a young age, which is consistent with previous studies showing a worse global systolic LS in GRMD dogs [8,10]. These results suggest that analysis of LS remains a sensitive tool to detect early alterations in LV systolic function in GRMD dogs.

A preferential alteration in subendocardial contractile function has been demonstrated in many pathological conditions such as exercise under acute increase in afterload [22], LV hypertrophy [23], coronary heart disease [24], and heart failure [25]. Previous studies have shown an early alteration of subendocardial systolic velocity analyzed by TDI in GRMD dogs [4,8]. This non-uniform change in subendocardial-subepicardial function has been considered to contribute to the progression of the disease. Interestingly, the present study showed that the left ventricle of GRMD dogs exhibits spatial non-uniform alteration in myocardial strain that occurs particularly in the apical region for CS. Because this non-uniform alteration in myocardial strain occurs early, it is reasonable to speculate that it is an important factor contributing to the progression of LV global contractile dysfunction.

The study may have some limitations. (1). Due to the obvious physical differences between GRMD and healthy control dogs, it is impossible for the investigator to be blind to the study group, which may lead to study bias. (2). This study characterized the temporal changes of LV myocardial strain in GRMD dogs but did not provide cellular and molecular mechanisms to explain this early change in myocardial strain. Thus, further studies of cellular and molecular mechanisms are needed. (3). The LV myocardial wall is a three-dimensional object and has strain that can occur along three planes. During contraction, the heart is not only shortening along its long and short axis, but also rotating, tilting and displacing inside the chest. Thus, in addition to technical factors that may influence the analysis of strain [12], this may also affect linear strain analyzed by 2D-STE, which is based on a simplistic sense of lengthening, shortening, thickening or rotating. In addition, the small thickness of the myocardial wall of young dogs (to be further divided into different layers by the software) and the high heart rate may increase the difficulty for accurate analysis of myocardial strain and possibly affect the accuracy of results. Therefore, caution should be taken when interpreting these results. However, the results obtained in young and adult dogs regardless of their conditions (healthy or GRMD) provide consistent myocardial strain information which is consistent with classic notions about LV contractile function. For example, both CS and LS data showed that the myocardial strain of the subendocardial layer is stronger than that in the subepicardial layer, which is consistent with the classic concept that the subendocardial layer contracts more strongly than the subepicardial layer. Further development of three-dimensional STE may give a more physiological description of LV myocardial strain reflecting the complexity of the heart’s three-dimensionality, and a recent study showed early impairment of LV 3-dimensional global LS in young patients with DMD [26].

## 5. Conclusions

In young dogs with DMD who have a normal global systolic function, a non-uniform reduction in systolic CS can be detected, especially in the LV apex, while decreased systolic LS can be detected in the three LV wall layers from three apical views. Our results suggest that analysis of myocardial strain by multi-layer 2D-STE may be a useful tool for monitoring the spatial evolution of alterations in LV systolic function and their progression with age in GRMD dogs. Given the similarity in pathogenesis, pathological changes, and clinical signs and symptoms between GRMD and DMD, our results suggest that assessing LV myocardial strain mechanics may be useful in detecting the early alteration of LV systolic function in early childhood of DMD patients and monitor its progression. The significance of these findings may warrant further investigation in DMD patients.

## Figures and Tables

**Table 1:** Body weight and LV dimensional and functional parameters measured by echocardiography in GRMD and healthy control dogs.

Parameter	Group Control GRMD	Age, Months						ANOVA *p* Value	
		2 n = 22 n = 7	6 n = 22 n = 7	9 n = 17 n = 4	12 n = 13 n = 4	18 n = 8 n = 4	24 n = 8 n = 4	Within-Group Effect	Between- Group Effect
body weight (kg)	Control	4.4 ± 0.4	20.1 ± 1.3	27.5 ± 2.2	29.5 ± 1.6	27.8 ± 1.6	26.2 ± 1.7	<0.0001	0.0003
	GRMD	3.1 ± 0.2 †	14.8 ± 0.7 †	18.3 ± 0.6 †	20.7 ± 0.9 †	20.5 ± 0.9 †	20.2 ± 0.9 †	<0.0001	
LV EDD (cm)	Control	2.3 ± 0.1	4.1 ± 0.2	4.4 ± 0.3	4.6 ± 0.3	4.4 ± 0.1	4.6 ± 0.3	<0.0001	0.0061
	GRMD	2.1 ± 0.1 *	3.5 ± 0.1 †	3.7 ± 0.1 *	4.2 ± 0.1	4.4 ± 0.1	4.5 ± 0.2	<0.0001	
LV ESD (cm)	Control	1.4 ± 0.1	2.5 ± 0.2	2.8 ± 0.2	3.0 ± 0.3	2.9 ± 0.2	2.9 ± 0.3	<0.0001	0.7718
	GRMD	1.3 ± 0.1	2.3 ± 0.1	2.5 ± 0.1	2.9 ± 0.1	3.1 ± 0.1	3.4 ± 0.2	<0.0001	
FS (%)	Control	37.4 ± 1.7	38.5 ± 0.9	35.3 ± 1.6	35.2 ± 1.6	35.2 ± 2.9	37.6 ± 2.5	0.5206	0.0015
	GRMD	38.3 ± 1.3	35.4 ± 1.1	32.8 ± 1.2	30.8 ± 1.1 *	29.6 ± 1.2 *	24.9 ± 1.6 †	<0.0001	
IVSWEDT (cm)	Control	0.49 ± 0.05	0.84 ± 0.06	1.00 ± 0.10	1.14 ± 0.10	1.12 ± 0.10	1.19 ± 0.11	<0.0001	0.0005
	GRMD	0.43 ± 0.01	0.70 ± 0.03 *	0.80 ± 0.03 †	0.71 ± 0.03 †	0.70 ± 0.04 †	0.73 ± 0.06 †	<0.0001	
IVSWTh (cm)	Control	0.29 ± 0.02	0.51 ± 0.04	0.64 ± 0.09	0.59 ± 0.08	0.52 ± 0.03	0.54 ± 0.01	<0.0001	0.0003
	GRMD	0.28 ± 0.02	0.45 ± 0.03	0.37 ± 0.03 †	0.42 ± 0.03 *	0.38 ± 0.04 *	0.28 ± 0.04 †	<0.0001	
%IVSWTh (%)	Control	62.5 ± 8.3	62.8 ± 6.3	65.2 ± 10.9	53.2 ± 10.1	48.1 ± 6.2	46.2 ± 4.4	0.3618	0.9705
	GRMD	67.1 ± 5.5	65.0 ± 4.0	46.4 ± 3.2 *	60.5 ± 4.9	54.7 ± 4.8	39.7 ± 6.4	0.0010	
PWEDT (cm)	Control	0.40 ± 0.02	0.75 ± 0.06	0.76 ± 0.03	0.93 ± 0.08	1.12 ± 0.10	1.08 ± 0.11	<0.0001	0.001
	GRMD	0.36 ± 0.03	0.59 ± 0.02	0.64 ± 0.03 *	0.73 ± 0.04 *	0.71 ± 0.06 *	0.73 ± 0.06 †	<0.0001	
PWTh (cm)	Control	0.30 ± 0.02	0.51 ± 0.04	0.57 ± 0.04	0.54 ± 0.04	0.60 ± 0.08	0.69 ± 0.06	<0.0001	0.0024
	GRMD	0.28 ± 0.01	0.36 ± 0.02 †	0.39 ± 0.03 *	0.36 ± 0.02 †	0.38 ± 0.05 *	0.28 ± 0.11 †	0.0028	
%PWTh (%)	Control	75.7 ± 8.9	71.0 ± 7.9	74.3 ± 4.9	58.7 ± 9.8	59.0 ± 7.6	65.1 ± 4.6	0.4770	0.0966
	GRMD	79.8 ± 4.8	61.7 ± 3.2	61.9 ± 6.0	51.3 ± 4.6	52.3 ± 5.4	40.3 ± 6.6 *	<0.0001	
Heart rate (beats/min)	Control	153.6 ± 5.5	98.4 ± 5.2	91.7 ± 2.4	81.7 ± 7.8	75.8 ± 5.1	83.1 ± 2.8	<0.0001	0.0751
	GRMD	161.0 ± 6.1	118.6 ± 3.5 †	108.2 ± 4.2	95.2 ± 3.6	102.1 ± 5.3 †	90.9 ± 6.6	<0.0001	
LV EDV (mL)	Control	10.8 ± 0.8	42.9 ± 5.3	63.4 ± 4.5	68.2 ± 5.8	76.2 ± 4.4	83.0 ± 7.8	<0.0001	0.0020
	GRMD	6.6 ± 0.4 †	30.8 ± 1.2 †	43.1 ± 2.5 †	49.5 ± 2.9 †	52.5 ± 3.3 †	61.5 ± 4.4 *	<0.0001	
LV ESV (mL)	Control	4.5 ± 0.4	15.9 ± 1.5	22.1 ± 1.7	27.2 ± 2.9	28.4 ± 2.8	34.2 ± 3.3	<0.0001	0.1450
	GRMD	2.7 ± 0.2 †	13.1 ± 0.5 *	20.1 ± 1.1	23.8 ± 2.0	26.5 ± 2.4	34.8 ± 3.0	<0.0001	
EF (%)	Control	58.8 ± 1.4	61.5 ± 3.0	64.3 ± 2.8	60.2 ± 1.8	63.1 ± 2.4	58.8 ± 0.5	0.4088	<0.0001
	GRMD	58.3 ± 1.2	57.3 ± 1.0	52.6 ± 1.3 †	52.5 ± 1.4 †	50.0 ± 2.0 †	43.8 ± 1.6 †	<0.0001	

Data were expressed as mean ± SEM. The one-way ANOVA was performed to test within-group differences. The ANOVA for repeated measurements over time was performed on echocardiographic data from 8 GRMD (golden retriever muscular dystrophy) and 4 healthy control dogs aged 2 to 24 months to test between-group differences. The two-tailed unpaired two-sample *t*-test was performed on data obtained in all dogs at each time point to determine the differences between GRMD and healthy control dogs. * *p* < 0.05 and † *p* < 0.01 versus healthy control dogs of the same age. EDD: end-diastolic diameter; EDV: end-diastolic volume; EF: ejection fraction; ESD: end-systolic diameter; ESV: end-systolic volume; FS: fractional shortening; IVSWEDT: interventricular septal wall end-diastolic thickness; IVSWTh: interventricular septal wall systolic thickening; % IVSWTh: percentage of interventricular septal wall systolic thickening; PWEDT: posterior wall end-diastolic thickness; LV: left ventricular; PWTh: posterior wall systolic thickening; % PWTh: percentage of posterior wall systolic thickening.

**Table 2:** Circumferential strain in myocardial layers of the left ventricle analyzed by 2-dimensional speckle tracking echocardiography in healthy control and GRMD dogs at different ages.

Parameter	Group Control GRMD	Age, Months						ANOVA *p* Value	
		2 n = 22 n = 7	6 n = 22 n = 7	9 n = 17 n = 4	12 n = 13 n = 4	18 n = 8 n = 4	24 n = 8 n = 4	Within−Group Effect	Between−Group Effect
Apical endocardial CS	Control	−41.2 ± 2.7	−37.4 ± 2.1	−38.8 ± 4.5	−34.5 ± 0.8	−33.2 ± 1.7	−34.7 ± 2.3	0.2656	0.0031
	GRMD	−35.1 ± 1.4 *	−35.5 ± 1.1	−34.4 ± 1.4	−31.1 ± 1.1	−26.5 ± 2.1	−24.8 ± 2.0 *	<0.0001	
Apical middle layer CS	Control	−27.7 ± 1.2	−25.5 ± 1.2	−25.6 ± 2.2	−22.5 ± 0.7	−23.4 ± 0.8	−22.9 ± 1.1	0.0626	0.0004
	GRMD	−21.9 ± 0.6 †	−22.8 ± 0.5 *	−22.9 ± 0.9	−20.7 ± 0.6	−17.7 ± 1.3 †	−17.4 ± 1.3 †	<0.0001	
Apical epicardial CS	Control	−18.2 ± 0.9	−17.6 ± 1.1	−18.0 ± 1.5	−15.0 ± 0.1	−16.5 ± 0.6	−14.7 ± 0.7	0.0881	0.0025
	GRMD	−14.8 ± 0.6 †	−14.3 ± 0.4 †	−14.8 ± 0.6 *	−13.3 ± 0.7	−11.7 ± 1.0 *	−12.2 ± 0.9	0.0054	
Mid−chamber endocardial CS	Control	−37.3 ± 0.8	−31.9 ± 1.1	−30.9 ± 1.1	−32.2 ± 3.0	−29.8 ± 1.3	−31.9 ± 2.6	0.0068	0.1544
	GRMD	−33.2 ± 0.7 †	−33.0 ± 0.8	−31.9 ± 1.2	−30.7 ± 1.2	−25.9 ± 1.8	−22.9 ± 1.9 *	<0.0001	
Mid−chamber middle layer CS	Control	−24.7 ± 0.8	−23.0 ± 0.9	−23.1 ± 0.8	−22.6 ± 1.2	−22.0 ± 0.8	−23.8 ± 1.3	0.2885	0.1028
	GRMD	−23.7 ± 0.5	−22.6 ± 0.5	−22.4 ± 0.9	−21.5 ± 0.7	−18.1 ± 0.7 †	−16.5 ± 0.8 †	<0.0001	
Mid−chamber epicardial CS	Control	−15.7 ± 1.0	−16.7 ± 0.9	−17.3 ± 1.0	−15.5 ± 0.0	−16.4 ± 0.9	−18.2 ± 0.6	0.3164	0.1393
	GRMD	−16.5 ± 0.5	−15.1 ± 0.5	−15.8 ± 0.8	−14.6 ± 0.6	−12.4 ± 0.5 †	−11.7 ± 0.5 †	<0.0001	
Basal endocardial CS	Control	−32.7 ± 1.5	−30.4 ± 1.0	−32.2 ± 1.5	−30.3 ± 2.5	−26.0 ± 1.7	−27.1 ± 1.6	0.0216	0.0406
	GRMD	−32.7 ± 0.9	−31.8 ± 1.1	−31.1 ± 1.5	−27.5 ± 1.4	−25.9 ± 1.5	−21.9 ± 1.3 *	<0.0001	
Basal middle layer CS	control	−23.1 ± 1.0	−21.8 ± 0.5	−24.2 ± 1.2	−22.6 ± 1.1	−20.6 ± 1.0	−19.8 ± 1.3	0.0383	0.0069
	GRMD	−23.0 ± 0.6	−22.5 ± 0.7	−22.0 ± 1.0	−21.0 ± 1.0	−18.1 ± 1.2	−17.0 ± 1.4 *	<0.0001	
Basal epicardial CS	Control	−16.7 ± 1.1	−15.6 ± 0.4	−18.4 ± 1.2	−16.7 ± 0.5	−17.0 ± 0.7	−14.6 ± 1.6	0.1231	0.0023
	GRMD	−16.2 ± 0.6	−16.0 ± 0.6	−15.2 ± 0.7 *	−16.5 ± 0.9	−13.1 ± 0.8 †	−13.6 ± 0.8 *	0.0139	

Data were expressed as mean ± SEM. The one-way ANOVA was used to test within-group differences over time (age). The ANOVA for repeated measurements over time was carried out on echocardiographic data obtained from 8 GRMD (golden retriever muscular dystrophy) dogs and 4 healthy control dogs aged 2 to 24 months to test between-group differences. The two-tailed unpaired two-sample *t*-test was performed on data obtained in all dogs to determine the differences between GRMD and healthy control dogs at each time point. * *p* < 0.05 and † *p* < 0.01 versus healthy control dogs of the same age. CS: circumferential strain.

**Table 3:** Longitudinal strain in myocardial layers of the left ventricle analyzed by 2-dimensional speckle tracking echocardiography in healthy control and GRMD dogs at different ages.

Parameter	Group Control GRMD	Age, Months						ANOVA *p* Value	
		2 n = 22 n = 7	6 n = 22 n = 7	9 n = 17 n = 4	12 n = 13 n = 4	18 n = 8 n = 4	24 n = 8 n = 4	Within−Group Effect	Between−Group Effect
3C endocardial LS	Control	−28.8 ± 1.9	−26.2 ± 1.7	−25.8 ± 0.9	−26.1 ± 0.8	−23.5 ± 2.4	−21.7 ± 1.1	0.0702	0.0010
	GRMD	−22.6 ± 0.6 †	−22.8 ± 0.8 *	−22.1 ± 1. 0 *	−21.9 ± 0.7 *	−22.1 ± 1.2	−19.5 ± 1.7	0.3076	
3C middle layer LS	Control	−24.0 ± 1.8	−22.8 ± 1.5	−22.3 ± 0.8	−22.9 ± 1.0	−20.3 ± 2.2	−18.3 ± 0.6	0.1366	0.0007
	GRMD	−18.9 ± 0.5 †	−19.2 ± 0.6 *	−19.0 ± 0.7 *	−18.7 ± 0.8 *	−18.9 ± 0.9	−16.7 ± 1.6	0.4422	
3C epicardial LS	Control	−19.9 ± 1.7	−19.9 ± 1.5	−19.3 ± 0.7	−20.1 ± 1.3	−17.6 ± 2.1	−15.5 ± 0.4	0.2452	0.0010
	GRMD	−15.9 ± 0.5 †	−16.3 ± 0.5 †	−16.5 ± 0.7	−16.2 ± 0.6 *	−16.2 ± 0.7	−14.4 ± 1.5	0.5367	
4C endocardial LS	Control	−26.3 ± 1.3	−25.7 ± 1.2	−27.9 ± 2.1	−25.4 ± 1.5	−24.3 ± 0.6	−21.4 ± 0.4	0.0881	0.0030
	GRMD	−23.5 ± 0.6 *	−22.9 ± 0.5 *	−22.5 ± 1.0 *	−21.7 ± 0.9	−21.7 ± 1.3	−16.8 ± 0.7 †	0.0002	
4C middle layer LS	Control	−22.8 ± 1.3	−22.3 ± 0.9	−23.7 ± 1.5	−21.6 ± 1.2	−20.3 ± 0.4	−18.7 ± 0.5	0.0863	0.0034
	GRMD	−20.0 ± 0.5 *	−19.6 ± 0.4 †	−19.1 ± 0.9 *	−18.5 ± 0.8	−18.7 ± 1.0	−14.4 ± 0.7 †	0.0002	
4C epicardial LS	Control	−20.8 ± 1.4	−19.6 ± 0.8	−20.1 ± 1.0	−18.4 ± 1.0	−17.0 ± 0.5	−16.3 ± 0.6	0.1023	0.0069
	GRMD	−17.0 ± 0.5 *	−16.9 ± 0.4 †	−16.2 ± 0.8 *	−16.0 ± 0.7	−16.2 ± 0.8	−12.5 ± 0.7 †	0.0006	
2C endocardial LS	Control	−26.0 ± 0.6	−25.9 ± 0.9	−25.2 ± 1.2	−25.6 ± 1.0	−22.6 ± 2.0	−22.1 ± 1.2	0.0277	0.0054
	GRMD	−23.2 ± 0.5 †	−22.6 ± 0.5 †	−21.8 ± 0.7 *	−20.0 ± 0.8 †	−19.7 ± 1.3	−19.4 ± 1.3	0.0006	
2C middle layer LS	Control	−22.7 ± 0.6	−22.2 ± 0.9	−21.5 ± 1.2	−21.7 ± 0.7	−19.7 ± 1.4	−19.6 ± 0.9	0.0746	0.0035
	GRMD	−19.5 ± 0.4 †	−19.6 ± 0.4 †	−19.0 ± 0.7	−17.4 ± 0.7 †	−17.6 ± 1.2	−16.7 ± 1.1	0.0088	
2C epicardial LS	Control	−20.1 ± 0.5	−19.1 ± 1.0	−18.4 ± 1.5	−18.3 ± 0.8	−17.2 ± 1.1	−17.5 ± 0.7	0.1672	0.0032
	GRMD	−16.4 ± 0.4 †	−17.2 ± 0.4 *	−16.8 ± 0.7	−15.3 ± 0.6 *	−15.8 ± 1.0	−14.4 ± 1.0	0.0398	

Data were expressed as mean ± SEM. The one-way ANOVA determined within-group differences over time (age). The ANOVA for repeated measurements over time was carried out on echocardiographic data obtained from 8 GRMD (golden retriever muscular dystrophy) dogs and 4 healthy control dogs aged 2 to 24 months to test between-group differences. The two-tailed unpaired two-sample *t*-test was performed on data obtained in all dogs to determine the differences between GRMD and healthy control dogs at each time point. * *p* < 0.05 and † *p* < 0.01 versus healthy control dogs of the same age. LS: longitudinal strain; 2C, 3C and 4C: 2-chamber, 3-chamber and 4-chamber.

## Data Availability

All relevant data generated in this study are available in this article.

## Abbreviations

CS: circumferential strain; DMD: Duchenne muscular dystrophy; EDD: end-diastolic diameter; EDV: end-diastolic volume; EF: ejection fraction; ESD: end-systolic diameter; ESV: end-systolic volume; FS: fractional shortening; GRMD: golden retriever muscular dystrophy; LS: longitudinal strain; LV: left ventricular; IVSWEDT: interventricular septal wall end-diastolic thickness; IVSWTh: interventricular septal wall systolic thickening; % IVSWTh: percentage of interventricular septal wall systolic thickening: PSAX: parasternal short-axis; PWEDT: posterior wall end-diastolic thickness; PWTh: posterior wall systolic thickening; % PWTh: percentage of posterior wall systolic thickening; STE: speckle tracking echocardiography; TDI: tissue Doppler imaging; 2C, 3C and 4C: 2-chamber, 3-chamber and 4-chamber.
